# A RNA sequencing-based six-gene signature for survival prediction in patients with glioblastoma

**DOI:** 10.1038/s41598-019-39273-4

**Published:** 2019-02-22

**Authors:** Shuguang Zuo, Xinhong Zhang, Liping Wang

**Affiliations:** 1 Center for Translational Medicine, Huaihe Hospital of Henan University, Kaifeng, Henan Province 475001 China; 2 Institute of Infection and Immunity, Huaihe Hospital of Henan University, Kaifeng, Henan Province 475001 China; 3 Zhengzhou Railway Vocational and Technical College, Zhengzhou, Henan Province 450052 China

## Abstract

Glioblastoma (GBM) is an aggressive tumor of the central nervous system that has poor prognosis despite extensive therapy. Therefore, it is essential to identify a gene expression-based signature for predicting GBM prognosis. The RNA sequencing data of GBM patients from the Chinese Glioma Genome Atlas (CGGA) and The Cancer Genome Atlas (TCGA) databases were employed in our study. The univariate and multivariate regression models were utilized to assess the relative contribution of each gene to survival prediction in both cohorts, and the common genes in two cohorts were identified as a final prognostic model. A prognostic risk score was calculated based on the prognostic gene signature. This prognostic signature stratified the patients into the low- and high-risk groups. Multivariate regression and stratification analyses were implemented to determine whether the gene signature was an independent prognostic factor. We identified a 6-gene signature through univariate and multivariate regression models. This prognostic signature stratified the patients into the low- and high-risk groups, implying improved and poor outcomes respectively. Multivariate regression and stratification analyses demonstrated that the predictive value of the 6-gene signature was independent of other clinical factors. This study highlights the significant implications of having a gene signature as a prognostic predictor in GBM, and its potential application in personalized therapy.

## Introduction

Glioblastoma (GBM) is the most common and aggressive tumor of the central nervous system in adults. Currently, standard therapy for newly diagnosed GBM is surgical resection to the viable extent, followed by radiotherapy and adjuvant chemotherapy^1,2^. Although the survival of GBM patients has improved due to advances in modern combination therapies, GBM still has the worst 5-year overall survival rates among all human cancers^3^, with a dismal median duration of 14 months^4,5^. Accumulating evidence in recent years shows that tumors consist of multiple cancer cell populations, each harboring specific genetic abnormalities^6^. Large-scale transcriptome studies on GBM have indicated the possible mechanisms underlying its aggressive behavior^7,8^, and substantial effort has focused on identifying those genetic alterations that might predict patient prognosis and therapeutic response^9^.

GBM is a highly heterogeneous disease consisting of multiple molecular alterations^7^. The differential molecular characteristics of histologically similar tumors make it challenging to predict the clinical outcomes and select the optimum treatment strategies. Currently, prognosis and risk stratification of GBM patients are largely based on the clinico-pathological features including age, tumor size, performance status and grade^10^. Given the heterogeneity of GBM and the multitude of factors influencing disease progression, conventional clinical characteristics are insufficient to accurately predict individual outcomes and survival. Therefore, it is necessary to identify different GBM specific genomic signatures to improve prognostic and therapeutic success.

The advances in sequencing and bioinformatics technologies have permitted genome-wide sequence analyses in many cancers including GBM. Sequencing data of a multitude of GBM samples have been archived in the open access databases, such as the Chinese Glioma Genome Atlas (CGGA, http://www.cgga.org.cn/) and The Cancer Genome Atlas (TCGA, https://portal.gdc.cancer.gov/). The aim of this study was to identify a prognostic signature of GBM using the RNAseq data of GBM patients from the CGGA and TCGA databases. A reliable genomic prognostic signature can complement the conventional clinical prognostic factors, and further enable personalized therapy.

## Results

### Identification of prognostic gene signature

**Figure 1:**
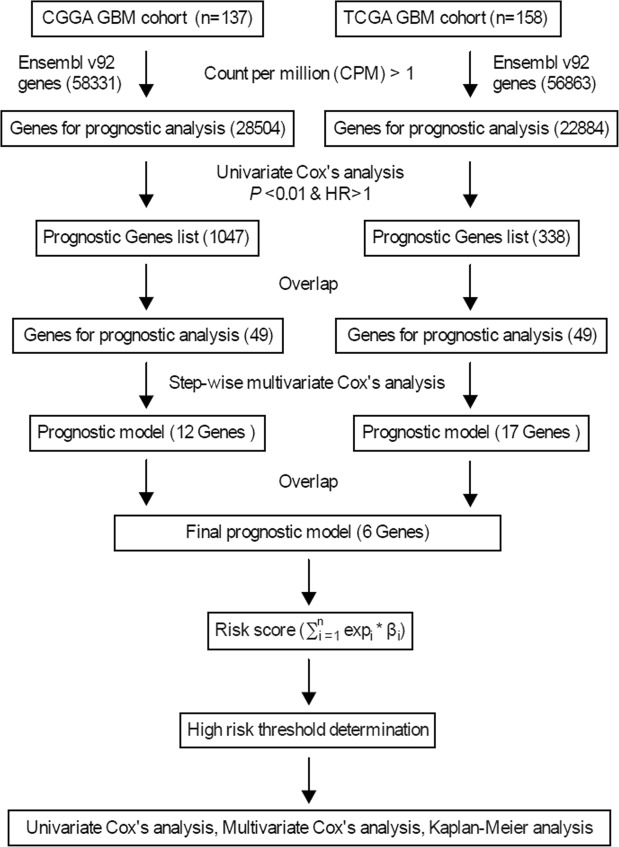
Study outline.

There were 58,331 and 56,863 genes in the CGGA and TCGA cohorts respectively, and after filtering, 28,504 and 22,884 genes were used for Cox’s regression analyses to screen for candidate genes associated with overall survival. Using the cut-off values of P < 0.01 and HR > 1, we identified 1047 and 338 candidate genes in the CGGA and TCGA cohorts respectively, of which 49 were common in both. Step-wise multivariate Cox’s regression analysis identified 12 and 17 genes respectively in the CGGA and TCGA cohorts that were independent survival predictors, of which CD79B, MAP2K3, IMPDH1, SLC16A3, MPZL3 and APOBR were common to both and thus subsequently were analyzed (Fig. 1). The 6 genes are summarized in Table 1.

**Table 1:** General information of 6 genes for constructing the prognostic signature.

Gene stable ID	Gene name	Gene type	Chromosome	Gene start (bp)	Gene end (bp)
ENSG00000007312	CD79B	protein_coding	17	63928740	63932354
ENSG00000034152	MAP2K3	protein_coding	17	21284672	21315240
ENSG00000106348	IMPDH1	protein_coding	7	128392277	128410252
ENSG00000141526	SLC16A3	protein_coding	17	82228397	82261129
ENSG00000160588	MPZL3	protein_coding	11	118226690	118252350
ENSG00000184730	APOBR	protein_coding	16	28494649	28498970

### Validation of the 6-gene signature in the two independent GBM cohorts

**Figure 2:**
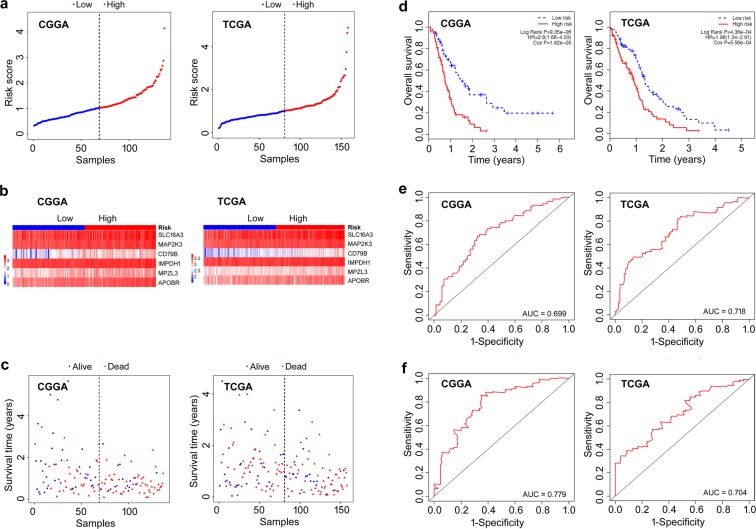
Correlation between the 6-gene signature and overall survival of patients. (**a**) The distribution of risk scores. (**b**) The expression heatmap of the 6 prognostic genes. (**c**) Scatterplot of patient survival status. (**d**) Kaplan-Meier curves of overall survival of the low- and high-risk groups. (**e**) ROC curve for 1-year survival prediction by the 6-gene signature. (**f**) ROC curve for 2-year survival prediction by the 6-gene signature. The black dotted line in a and c represents the median risk score cutoff dividing patients into the low- and high-risk groups.

**Figure 3:**
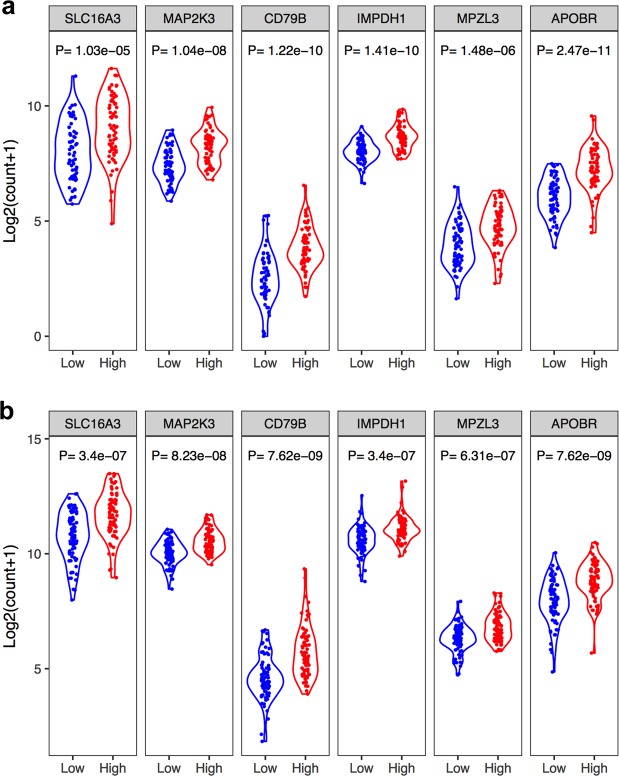
The expression levels of the individual genes of the 6-gene signature. Violin plot showing the expression levels of the genes between low- and high-risk groups in the CGGA cohort (**a**) and TCGA cohort (**b**).

Based on the expression levels of the 6 survival-relevant genes and their relative contributions as per the multivariate regression analysis, we developed a prognostic model by the risk score method for survival prediction. This 6-gene signature-based prognostic model endowed a risk score for each GBM patient, and was validated in the CGGA and TCGA cohorts. In both the CGGA and TCGA cohorts, patients were divided into the low- and high-risk groups using the median risk score as cutoff threshold. The distribution of risk scores, the expression values of the six genes and the survival status of patients ranked according to the risk scores are shown in Fig. 2a–c. Kaplan-Meier survival curves with log-rank test showed that patients in the high-risk group had a significantly shorter survival duration compared to the low-risk group (Fig. 2d, log-rank P = 9.35e-06 for CGGA cohort; log-rank P = 4.36e-04 for TCGA cohort). High risk score was therefore an adverse prognostic factor for GBM patients (HR = 2.6, 95% CI = 1.68–4.03 for CGGA cohort; HR = 1.98, 95% CI = 1.34–2.91 for TCGA cohort). The 1-year and 2-year survival as predicted by the risk scores are shown in Fig. 2e,f, with AUC values of 0.699 and 0.779 for CGGA cohort, 0.718 and 0.704 for TCGA cohort, respectively, implying that the 6-gene signature had high specificity and sensitivity in predicting survival, and was competent for predicting the survival of GBM patients. Finally, the expression levels of all the six genes were significantly higher in the high-risk compared to the low-risk groups in both cohorts (Fig. 3).

### The 6-gene signature is an independent prognostic factor of survival

Cox’s regression models were used to determine whether the prognostic value of the 6-gene signature was independent of other clinical factors in each cohort. The results are shown in Table 2. Univariate regression analysis of the CGGA cohort indicated that the 6-gene signature-based risk score (HR = 2.60, 95% CI = 1.68–4.03; P = 1.82e-05), radiation therapy (HR = 0.41, 95% CI = 0.26–0.66; P = 1.85e-04), MGMT expression (HR = 1.37, 95% CI = 1.12–1.68; P = 2.19e-03) and chemotherapy (HR = 0.34, 95% CI = 0.21–0.53; P = 2.30e-06) were significantly associated with patient prognosis, while age, gender and other gene mutations showed no significant association with overall survival (P > 0.05). Multivariate regression analysis showed a significant correlation of the 6-gene signature with survival after adjusting for other clinical factors. The 6-gene risk score (HR = 2.40, 95% CI = 1.42–4.06; P = 1.11e-03) was an independent adverse prognostic factor, while radiation therapy (HR = 0.42, 95% CI = 0.25–0.69; P = 7.13e-04) and chemotherapy (HR = 0.35, 95% CI = 0.20–0.59; P = 8.50e-05) were independent favorable factors.

**Table 2:** Univariate and multivariate Cox’s regression analyses of the gene signature in two independent cohorts.

Variables		Patients (N)	Univariate analysis		Multivariate analysis	
			HR (95% CI)	*P*	HR (95% CI)	*P*
**CGGA**						
Age	<= 49/ > 49	73/64	1.00 (0.99–1.02)	5.76e-01	0.99 (0.96–1.02)	4.06e-01
Gender	Female/Male	47/90	1.22 (0.79–1.88)	3.75e-01	1.59 (0.88–2.86)	1.23e-01
Radiation therapy	No/Yes	44/79	0.41 (0.26–0.66)	1.85e-04	0.42 (0.25–0.69)	7.13e-04
Chemotherapy	No/Yes	41/82	0.34 (0.21–0.53)	2.30e-06	0.35 (0.20–0.59)	8.50e-05
IDH1 mutation	No/Yes	104/33	0.64 (0.38–1.07)	8.57e-02	1.24 (0.54–2.83)	6.16e-01
TP53 mutation	No/Yes	62/75	0.70 (0.46–1.05)	8.64e-02	0.87 (0.54–1.41)	5.72e-01
EGFR mutation	No/Yes	101/36	0.74 (0.46–1.21)	2.34e-01	0.57 (0.31–1.03)	6.64e-02
ATRX mutation	No/Yes	125/12	0.79 (0.38–1.63)	5.17e-01	0.76 (0.26–2.28)	6.28e-01
EZH2 mutation	No/Yes	115/22	1.13 (0.65–1.98)	6.68e-01	1.57 (0.81–3.06)	1.84e-01
MGMT expression	Low/High	69/68	1.37 (1.12–1.68)	2.19E-03	1.23 (0.94–1.62)	1.30E-01
Risk score	Low/High	69/68	2.60 (1.68–4.03)	1.82e-05	2.40 (1.42–4.06)	1.11e-03
**TCGA**						
Age	<= 60/ > 60	80/78	1.03 (1.01–1.04)	1.26e-03	1.01 (0.99–1.03)	2.33e-01
Gender	Female/Male	56/102	0.95 (0.65–1.41)	8.08e-01	1.22 (0.77–1.95)	4.03e-01
Radiation therapy	No/Yes	21/130	0.16 (0.09–0.26)	1.34e-12	0.12 (0.06–0.23)	7.76e-11
MGMT expression	Low/High	79/79	1.20 (1.01–1.43)	3.81E-02	1.26 (1.05–1.51)	1.10E-02
Risk score	Low/High	81/77	1.98 (1.34–2.91)	5.56e-04	1.70 (1.10–2.63)	1.73e-02

In the TCGA cohort, univariate analysis showed that age (HR = 1.03, 95% CI = 1.01–1.04; P = 1.26e-03), 6-gene risk score (HR = 1.98, 95% CI = 1.34–2.91; P = 5.56e-04), MGMT expression (HR = 1.20, 95% CI = 1.01–1.43; P = 3.81e-02), and radiation therapy (HR = 0.16, 95% CI = 0.09–0.26; P = 1.34e-12) were significantly associated with overall survival, while gender had no significant correlation (P > 0.05). Multivariate Cox’s regression analysis showed that age was not an independent factor. Furthermore, the 6-gene signature had a significant prognostic value after adjusting for the other clinical factors. Taken together, the 6-gene risk score (HR = 1.70, 95% CI = 1.10–2.63; P = 1.73e-02), and MGMT expression (HR = 1.26, 95% CI = 1.05–1.51; P = 1.10e-02) were independent adverse prognostic factors, while radiation therapy (HR = 0.12, 95% CI = 0.06–0.23; P = 7.76e-11) was an independent favorable factor.

### Stratification analysis: prognostic value of 6-gene signature stratified by clinical factors

**Figure 4:**
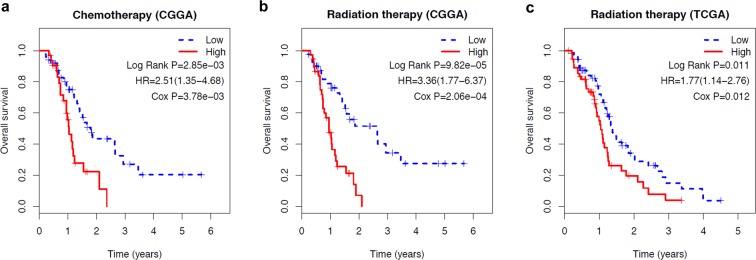
Kaplan-Meier analysis of overall survival of patients stratified by treatment. (**a**) Chemotherapy stratification analysis in CGGA cohort. (**b**) Radiation therapy stratification analysis in CGGA cohort. c: Radiation therapy stratification analysis in TCGA cohort.

**Figure 5:**
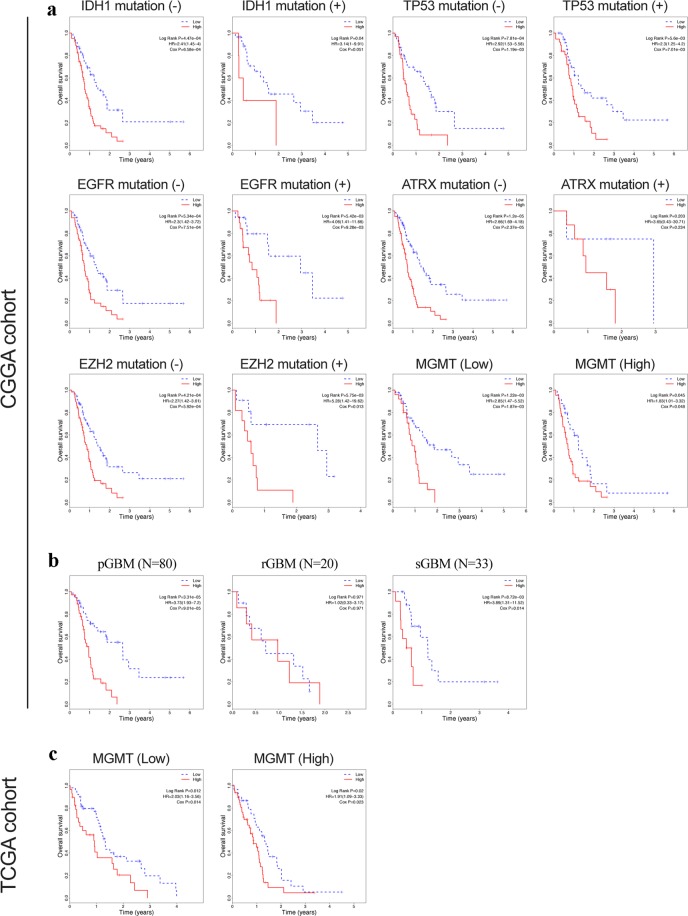
Kaplan-Meier analysis of overall survival of patients stratified by gene mutations or different GBM types. (**a**) Stratification analysis in CGGA cohort based on gene mutations **(**IDH1, TP53, EGFR, ATRX, EZH2, and MGMT**)**. (**b**) Stratification analysis for primary, secondary, and recurrent GBMs in CGGA cohort. (**c**) Stratification analysis in TCGA cohort based on MGMT mutations.

In this study, radiation therapy, chemotherapy, and MGMT expression were identified as survival-associated factors. Therefore, patients were further stratified on the basis of whether they received radio-/chemotherapy and the MGMT expression level, in order to assess the prognostic value of the 6-gene signature. The patients in each cohort were first stratified into subgroups (such as chemotherapy (no/yes), radiation therapy (no/yes), and MGMT expression (low/high)), and each subgroup was further divided into the low- and high-risk groups using the 6-gene signature. In the radio-/chemotherapy subgroups of the CGGA cohort, patients in the high-risk group had a significantly shorter survival duration compared to those in the low-risk group (Fig. 4a,b, P < 0.05), indicating that the 6-gene risk score was an adverse prognostic factor and could predict the survival of patients receiving chemotherapy (HR = 2.51, 95% CI = 1.35–4.68) or radiotherapy (HR = 3.36, 95% CI = 1.77–6.37). In the radiation therapy and MGMT expression subgroups of the TCGA cohort, patients in the high-risk group had significantly poorer prognosis than those in the low-risk group (Fig. 4c, P < 0.05 for radiation therapy and Fig. 5c, P < 0.05 for MGMT expression), further validating that the 6-gene risk score can predict survival in patients receiving radiation therapy (HR = 1.77, 95% CI = 1.14–2.76) or with high/low MGMT expression.

Then, we analyzed the relationship of risk score with gene mutations including IDH1, TP53, EGFR, ATRX, EZH2, and MGMT. To begin with, the patients in the CGGA cohort were firstly divided into two subgroups based on whether these gene were mutated or not, and next each subgroup was further separated into high- and low-risk group relying on the 6-gene signature. In the subgroups (except ATRX subgroup), patients in the high-risk group had a significantly worse prognosis, relative to the low-risk group (Fig. 5a, P < 0.05), implicating that the 6-gene risk score can predict the survival status in GBM patients.

Taken together, our findings suggested that the prognostic value of the 6-gene signature was independent of other clinical features for predicting survival in GBM patients.

### Comparison of selected genes expression between primary and secondary GBM

Only primary GBM data are included in the TCGA database, and there are primary GBM data, recurrent GBM data, and secondary data in the CGGA database. In our analysis, we used all GBM cases to establish a prognosis signature. However, primary and secondary GBMs are different diseases from the molecular point of view. Thus, we performed the grouping analysis for primary, secondary, and recurrent GBM data. The analysis results (Fig. 5b) show that this 6-gene signature can be used as a prognostic indicator for primary and secondary GBM. However, for recurrent GBM patients, there is no difference in survival in different risk score groups, suggesting that this 6-gene prognosis signature might not be applicable for recurrent GBM.

## Discussion

Studies show that gene alterations play significant roles in tumorigenesis and patient prognosis, indicating a potential application of characteristic gene signatures in cancer diagnosis and prognosis. Several reports are available on the correlation between altered genes and GBM prognosis. For example, Nicolasjilwan *et al*.^11^ analyzed the genetic biomarkers to predict the survival of GBM patients in the TCGA database based on clinical factors and imaging characteristics, but the sensitivity and specificity of the gene signature in survival prediction were not measured. Another study also using TCGA GBM data to detect an inverse correlation between IL-13Rα1 and α2 expression and unsatisfied prognosis^12^. Moreover, Jia *et al*.^13^ have identified prognosis-related genes for GBM based on TCGA and CGGA databases. However, these studies have focused on single GBM-related genes, which have limited prognostic and predicative power. Therefore, we tested the predictive power of a panel of GBM-associated genes using regression analyses. We developed a risk score model based on 6 genes to predict clinical outcomes of GBM patients by analyzing the RNAseq data from two open access databases. The 6-gene signature competitively predicted patient survival, and according to the univariate and multivariate analyses, was an independent prognostic factor in addition to chemotherapy therapy, radiation therapy, and MGMT expression.

Due to extensive heterogeneity of GBM, even patients with similar histo-pathological characteristics differ in their genetic landscape, making it challenging to effectively target different patients using the same protocol. Therefore, individualized risk stratification and treatment are urgently needed. Omics-based patient-specific therapy is based on the genomics, transcriptomics and proteomics data of individual patients. An understanding of patient-specific mutations would help design the most effective therapeutic strategy for that particular patient. Omics-based techniques can be used at both pre- and post-treatment stages to track specific mutations as well as development of resistance, and help modify the treatment accordingly.

Univariate analysis showed that age was not significantly associated with overall survival in the CGGA cohort, while a significant association was observed in the TCGA cohort. Age at diagnosis is a major predictor of patient survival, and younger patients tend to survive longer than the older patients^8,14,15^. However, age alone is not a predictor for survival in GBM because older patients are less likely to receive adjuvant treatment^16^. Multivariate analysis confirmed that age was not an independent predictor for survival in our cohorts as well. Currently, surgical resection, radiotherapy and adjuvant chemotherapy are the standard treatment options in GBM patients. For elderly patients, radiotherapy can improve survival without reducing life quality or cognition^17^. However, the survival is significantly reduced if radiotherapy is not initiated within 6 weeks after complete resection^18^. Furthermore, radiotherapy plus temozolomide provides a significant survival benefit compared to radiotherapy alone in treating GBM^19,20^. We also found that radiation therapy and chemotherapy were independent favorable prognostic factors. The 6-gene signature stratified the treated and untreated patients into the low- and high-risk groups that had significantly different prognosis, indicating that the 6-gene signature can improve survival prediction and can also identify high-risk patients for adjuvant therapy in addition to the standard regimen.

Several gene mutations have been identified which correlate with the pathogenesis of GBM^21^. EGFR mutations confer enhanced tumorigenicity by increasing proliferation and reducing apoptosis of GBM cells, especially in secondary GBM^22^. Mutations in IDH1 predict a more favorable prognosis^23–25^, and are often associated with TP53 and ATRX mutations^26,27^. The CGGA database includes gene mutations and gene expression profiles from a number of GBM patients. However, univariate and multivariate analyses showed that these mutations had no significant prognostic value, possibly because most of them are more common in patients with secondary GBM while most subjects in our study had primary GBM.

The results about the relationship of risk score with IDH1 mutations showed patients in the high-risk group had a significantly worse prognosis, relative to the low-risk group, which implicated that the 6-gene risk score is an independent prognostic factor independent of IDH1 mutation.

As we all know, MGMT promoter methylation ultimately determines the expression of MGMT, thus, we analyzed the relationship of prognosis and MGMT expression level in the CGGA and TCGA databases. Multivariate analysis exhibited that in the CGGA database, the expression of MGMT was not an independent prognostic factor, while MGMT was an independent prognostic factor in the TCGA database. The subgroup analysis based on MGMT expression level to reveal the relation between our risk score and MGMT expression showed that patients in the high-risk group had a significantly worse prognosis, relative to the low-risk group, which demonstrated that the 6-gene risk score is an independent prognostic factor independent of MGMT expression.

Our 6 prognostic panel genes have established roles in oncogenesis. For example, CD79B has been associated with reduced survival, and is considered a suitable immunotherapeutic target for leukemia and lymphoma^28,29^. Several studies have highlighted the critical role for MAP2K3 in tumor progression and targeted therapies^30,31^. MAP2K3 inhibition sensitizes tumor cells to chemotherapy^31^, and has been identified as a putative target for molecular therapy against GBM^32^. SLC16A3 is typically over-expressed in GBM and patients with higher SLC16A3 levels have significantly poorer survival^33,34^, making it a potential prognostic biomarker and metabolic target in GBM^35^. Based on the genomic locations for three genes (CD79B, MAP2K3, SLC16A3), we find that CD79B locates at chromosome 17q23.3, MAP2K3 locates at chromosome 17q11.2, and SLC16A3 locates at chromosome 17q25.3. Although these all locate on chromosome 17, these 3 genes are not close to one another. Chromosome 17, as one of 23 pairs of human chromosomes, whose abnormities and roles that have been investigated about the expression of genes in this chromosome influence the nervous system, especially cell differentiation and maturation. Deletions in the region of chromosome 17 are the most common abnormal event in primary solid tumors^36^. Petitjean *et al*.^37^ have suggested that the deletion of the region of chromosome 17 is greatly important in carcinogenesis, as found in other examples including breast cancer, colon cancer, liver cancer, medulloblastoma and in head tumors^36,38^. Further, Sunpaweravong *et al*.^39^ have implicated that gains of cytoband 17q25.3 are found in 61% lung cancer patients, underscoring a potential biological role for the genes within this region in the progression of lung cancer, which further substantiates the fact that there are many oncogenes located at chromosome 17. Significantly, loss of heterozygosity for the short arm of chromosome 17 has been demonstrated in a 4-year-old boy with GBM^40^. According to the importance of chromosome 17 described above, losses or changes in genes in chromosome 17 might potentially involve in GBM. In our study, it is more convinced that CD79B, MAP2K3, and SLC16A3 on chromosome 17 play important roles in GBM.

IMPDH1, one isotype of IMPDH, is ubiquitously expressed in mammals^41^. IMPDH1 up-regulation has been found in many types of cancer, such as bladder cancer, brain cancer, lung cancer, ovarian cancer, and GBM^42^. Increased MPZL3 is found to exert important function in ovarian cancer via regulating metablolism^43^. Moreover, RNA expression of MPZL3 has been reported to be highly expressed in radio-resistant rectal cancer cell lines^44^. APOBR mutation is observed in hepatocellular carcinoma^45^. In our study, this 6-gene signature would independently predict overall survival in GBM patients. The advantage of this work was to combine the clinical characteristics and CGGC/TCGA data to assess the GBM patients survival through establishing a genes-associated risk score. Therefore, the combination of these 6 genes (CD79B, MAP2K3, IMPDH1, SLC16A3, MPZL3 and APOBR) could be regarded as a novel risk factor that might function as a prognosis indicator for GBM patients.

To summarize, we combined 6 GBM specific genes into a single diagnostic panel using regression analyses, and established its predictive value in overall survival of the patients. We selected only those survival-related genes that were common to both the CGGA and TCGA cohorts, which is more reliable and stable relative to single cohort analysis.

In conclusion, we identified a 6-gene signature for predicting survival in GBM by analyzing RNAseq-based gene expression profiles in the CGGA and TCGA patients. Multivariate and stratified analysis demonstrated that the gene panel was independent of other clinical and pathological features, and therefore is a potential prognostic biomarker of GBM.

## Methods

### Patients

The transcriptomic data of GBM patients were obtained from two independent data portals (CGGA and TCGA). As part of the CGGA project, Zhao *et al*.^46^ provided the RNA-seq transcriptomic profiles of 325 gliomas samples. The raw RNA-seq data were downloaded from the NCBI Sequence Read Archive (SRA, https://www.ncbi.nlm.nih.gov/sra) using the accession numbers SRP027383 and SRP091303. The raw fastq files were converted from SRA using Fastq-dump app (version: 2.8.1), and the sam and read count data were obtained by the hisat2 (version 2.1.0) and htseq software (version 0.10.0), respectively. A total of 137 GBM samples with clinical data were selected from the CGGA cohort. Data of an independent cohort of 158 patients was obtained from the TCGA database. The transcriptomic profiles (level 3 data) and corresponding clinical data of these patients were downloaded from the Data Coordinating Center. All RNA-seq libraries were sequenced using the Illumina HiSeq2000 Systems. The raw count data were normalized by the trimmed mean of M-values (TMM) method^47^, and genes with extremely low total abundance (count per million <1) were filtered out. The data preprocessing was performed using edgeR package^48^ (version 3.20.9) in Bioconductor.

### Screening for the prognostic gene signature

The schematics of our study are illustrated in Fig. 1. Univariate Cox’s proportional hazards regression analysis was used to evaluate the correlation between the expression level of each gene and overall survival in both cohorts. Only genes with P values < 0.01 and hazard ratio (HR) > 1 were considered as candidate genes for the correlation analysis, and those common genes to both CGGA and TCGA cohorts were used to construct the predictive model. The common candidate genes were then fitted in step-wise multivariate Cox’s regression model to assess the relative contribution of each gene to survival prediction in both cohorts. The genes that correlated with survival and were common to both cohorts were included in the prognostic signature. According to the estimated regression coefficients in multivariate Cox’s regression model, a prognostic risk score for predicting overall survival was then calculated as follows,

$$Risk\,score=\sum _{i=1}^{n}ex{p}_{i}\ast {\beta }_{i}$$

where n is the number of prognostic genes, exp_i_ is the expression level of prognostic gene i, and β_i_ is the regression coefficient of gene i. Using the median risk score in CGGA cohort as the cutoff value, GBM patients in each cohort were divided into low- and high-risk groups.

### Statistical analysis

Kaplan-Meier analysis was used to determine the survival in the low- and high-risk groups in each cohort, and log-rank test was used to assess the statistical significance. Multivariate Cox’s regression model and stratification analysis were implemented to determine whether the gene signature was independent of other clinical features. The time-dependent receiver operating characteristic (ROC) curve was used to compare the sensitivity and specificity of the gene signature in survival prediction. The area under the curve (AUC) was calculated from the ROC curve. For both log-rank test and Cox regression analysis, the significance was set at P value < 0.05.
